# Comparisons of Ocular Anatomic Differences of Lens-Subluxated Eye with or without Acute Angle Closure: A Retrospective Study

**DOI:** 10.1155/2020/6974202

**Published:** 2020-07-30

**Authors:** Hong-Liang Lin, Yong-Jie Qin, Yu-Lin Zhang, Yu-Qiao Zhang, Yong-Yi Niu, Yan-Lei Chen, Yun-Yan Hu, Wen-Juan Xie, Hong-Yang Zhang

**Affiliations:** ^1^Department of Ophthalmology, Guangdong Eye Institute, Guangdong Provincial People's Hospital and Guangdong Academy of Medical Sciences, Guangzhou, Guangdong 510080, China; ^2^Shantou University Medical College, Shantou, Guangdong, China; ^3^Southern Medical University, Guangzhou, Guangdong, China

## Abstract

**Purpose:**

To compare ocular anatomy differences of lens subluxation between eyes with or without acute angle closure (AAC).

**Methods:**

This is a retrospective and case-control study. Sixty cases with mild lens subluxation were recruited. Among them, 30 eyes with acute angle closure were assigned to the AAC group and 30 eyes without AAC were assigned to the non-AAC group. The anterior segment was quantitatively evaluated by ultrasound biomicroscopy (UBM). The axial length (AL) was measured with IOL Master. All patients underwent lens extraction surgery and were followed up for six months.

**Results:**

The history of blunt trauma accounted for 22 (73.3%) cases in the AAC group and 21 (70%) cases in the non-AAC group. Fifteen (50%) patients in the AAC group had iridotomy history, and high intraocular pressure recurred. The UBM analysis showed that the average central chamber depth of the affected eyes in the AAC group was 1.82 mm, which was significantly shallower than that in the fellow eyes (2.58 mm, *P* < 0.05) or both eyes in the non-AAC group.Both eyes in the AAC group presented a shorter AL and shallower anterior chamber than the eyes in the non-AAC group.

**Conclusions:**

An asymmetrical anterior chamber between bilateral eyes is an important feature in lens subluxation-induced AAC. The crowded anterior chamber and shorter AL might be the anatomic basis for the eye with lens subluxation-induced AAC.

## 1. Introduction

Many conditions can result in lens subluxation, including congenital, developmental, traumatic, and iatrogenic zonulysis. The signs of lens subluxation include iridodonesis, phacodonesis, visibility of the lens equator, decentration of the lens, and vitreous prolapse in the anterior chamber, but not all patients with lens subluxation have the obvious signs mentioned earlier. Lens subluxation will cause the movement of the lens forward or backward. When the lens moves forward, it can lead to a decreased depth of the anterior chamber, even pupillary block, anterior chamber angle closure, or acute intraocular pressure rise [1, 2]. For the eyes with AAC caused by lens subluxation, having no clinical signs of the zonular weakness, it is difficult to determine the zonular stability before surgery and these signs may be neglected in the clinical setting [3]. Zonular dehiscence may be observed after full dilation of the pupil by slit-lamp examination, but that entails the risk of incidental IOP elevation [4]. Anteriorly positioned lens plays an important role in the acute angle closure caused by lens subluxation, but not all lens subluxation can cause acute angle closure. Except for an anteriorly positioned lens, differentiating the ocular anatomic difference is helpful in precisely diagnosing the eyes with lens subluxation-induced AAC. The present study was conducted to compare the ocular differences of lens subluxation between the eyes with or without AAC.

## 2. Materials and Methods

### 2.1. Subjects

We retrospectively reviewed 60 patients with unilateral mild lens subluxation who were recruited from the Department of Ophthalmology, Guangdong Provincial People's Hospital, from January 2016 to June 2019. All patients were divided into two groups, according to those with or without acute angle closure attack history. Thirty eyes of 30 patients with lens subluxation and acute angle closure were assigned to the AAC group. Thirty eyes of 30 patients with lens subluxation without acute angle closure history were assigned to the non-AAC group. Informed consent was obtained from each patient before surgery. This study was approved by the institutional Human Research Ethics Committee of Guangdong Provincial People's Hospital and Guangdong Academy of Medical Sciences, Guangzhou, China, and adhered to the tenets of the Declaration of Helsinki from the World Medical Association.

### 2.2. Inclusion Criteria and Exclusion Criteria

Only patients with mild lens subluxation and cataract were recruited in the study. The degree of lens dislocation in non-AAC group and AAC group was evaluated during cataract surgery. The classification method was described previously [5]: (1) minimal to mild lens subluxation in which the lens edge uncovers 0% to 25% of the dilated pupil; (2) moderate lens subluxation in which the lens edge uncovers 25% to 50% of the dilated pupil; and (3) severe lens subluxation in which the lens edge uncovers more than 50% of the dilated pupil.

Acute angle closure was defined as [6, 7] (1) presence of at least two of the following symptoms: ocular or periocular pain; nausea and/or vomiting; and a history of intermittent blurring of vision with haloes; (2) presenting with IOP higher than 21 mmHg in a Goldmann applanation tonometry test; (3) presence of at least three of following signs: conjunctival injection, corneal epithelial edema, middilated unreactive pupil, and shallow anterior chamber; and (4) presence of angle closure in the gonioscopy investigation. Patients with mild lens subluxation but without history or sign of previous acute angle closure were assigned to the non-AAC group.

Patients with the following conditions were excluded: (1) patients who have been diagnosed with congenital diseases relating to lens subluxation, such as Marfan syndrome; (2) patients who have been definitely diagnosed with primary AAC; (3) patients who have moderate or severe lens dislocation in the slit-lamp investigation; and (4) patients who are unable to cooperate with the ultrasound biomicroscopy (UBM) investigation. All patients were followed up for at least 6 months.

### 2.3. Examinations and Treatments

The medical history for each patient was recorded. These histories included age, gender, family medical history, metabolic and genetic syndromes, ocular trauma, date of onset of symptoms, diagnosis, and treatments. All subjects underwent comprehensive ophthalmic examinations, including assessment by Goldmann applanation tonometry, refraction tests, slit-lamp microscopy, gonioscopy, stereoscopic examination of the optic disc, and optic coherence tomography (OCT) (Spectralis OCT, Heidelberg Engineering, Germany). Uncorrected and corrected distance visual acuities (UDVA and CDVA) were measured using the Snellen chart in decimal values. Gonioscopy, performed in a dark room (with and without indentation), was performed on all participants by a single examiner (ZHY), who was masked to the UBM findings, using a Goldmann two-mirror lens (Ocular Instruments Inc., Bellevue, WA, USA). The angle in each quadrant was graded based on the observed anatomical structures, following the modified Shaffer grading system [8, 9] (grade 0, no structure or Schwalbe's line observed; grade 1, visible anterior nonpigmented trabecular meshwork; grade 2, visible posterior pigmented trabecular meshwork; grade 3, visible scleral spur; grade 4, visible ciliary body). Angle quadrants were considered closed if they were grade 0 or 1. Gonioscopic angle closure in an eye was defined as closure in two or more quadrants [10]. Quantitative parameters of the anterior segment, including the angle opening distance at 500 *μ*m from the scleral spur (AOD 500), anterior chamber depth (ACD), lens vault (LV), iris thickness at 750 *μ*m from the scleral spur (IT 750), iris curvature (I-curve), and the status of the iris and zonules, were measured by ultrasound biomicroscopy (UBM SW-3200L, Suoer Electrotec AG, China). In addition, the axial length (AL) was measured by IOL Master 500 (Carl Zeiss Meditec AG, Germany). These procedures were performed by the same experienced ophthalmic technician (WJX). Qualitative UBM diagnoses were independently provided by two glaucoma ophthalmologists (XJX and YJQ). Preoperative IOL power calculations were performed using the AL and the keratometry readings measured by IOL Master. Phacoemulsification and implantation of spherical intraocular lenses (IOLs) with different diopters (Sensar AR40e, Abbott Medical Optics, CA, USA) were performed uneventfully by a single surgeon (HYZ). No complications occurred during the follow-up of 6 months.

### 2.4. Statistical Analysis

Descriptive statistics were calculated for the demographic characteristics of the AAC group and the non-AAC group. To compare the means between two groups, parametric variables were analyzed by using Student's *t*-test. Analyses of nonparametric variables were calculated with the Mann–Whitney *U* test. A *P*-value less than 0.05 was considered statistically significant. All analyses were conducted by using SPSS version 20.0 (SPSS Inc., Chicago, Illinois, USA).

## 3. Results

### 3.1. Demographic Data

In total, 60 eyes from 60 patients with lens subluxation were included in this study, and the features of the patients' histories are summarized in Table 1. The age, gender, and history of blunt ocular trauma were not significantly different between the AAC group and the non-AAC group (*P* > 0.05). The duration since ocular trauma was significantly longer in patients with a history of AAC attack than in the non-AAC group (*P*=0.001). Among the patients who suffered from acute angle closure in their first attack, 20 (66.7%) with lens subluxation-induced AAC were misdiagnosed with primary AAC. Fifteen (50%) patients in the AAC group had laser peripheral iridotomy (LPI) or surgical peripheral iridotomy (SPI) treatment history, and high intraocular pressure recurred. Before presenting to our department, all patients with AAC had received treatment with antiglaucoma medications for control of IOP. After removing the subluxated lens, the IOP of the patients was controlled without use of antiglaucoma medications. As shown in Table 2, the elevated IOP in the AAC group decreased significantly from preoperative (pre-op) 41.93 ± 13.64 mmHg to postoperative (post-op) 14.49 ± 6.03 mmHg (*P* < 0.001). The preoperative and postoperative IOP of the affected eyes in the non-AAC group were in the normal range (14.88 ± 5.32 versus 14.31 ± 4.63, *P*=0.310).

### 3.2. Biometric Features of Lens Subluxation in the AAC Group and the Non-AAC Group

**Figure 1:**
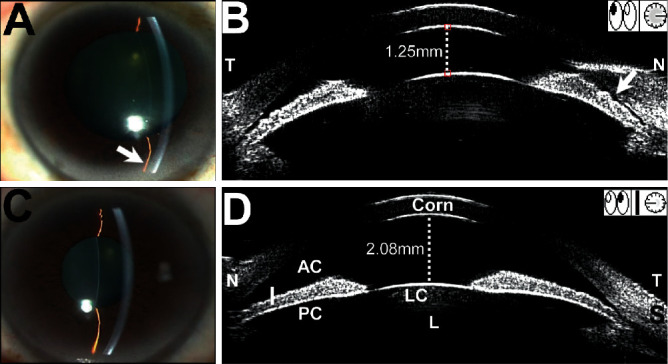
Clinical characteristics of a patient with lens subluxation with AAC attack. (a) Affected eye (right eye): slit-lamp biomicroscopy demonstrated that the anterior chamber depth (ACD) was shallow in central region, and it was shallower in periphery (arrow). Imaging by ultrasound biomicroscopy (UBM) showed a 1.25 mm central ACD, whereas the peripheral nasal ACD was shallower (arrows). The lens vault (LV) was 1.48 mm. (b) Fellow eye (left eye): the central and peripheral ACD were wider than in the affected eye. The central ACD was 2.08 mm and the lens vault (LV) was 0.95 mm. The LV is defined as the perpendicular distance between the anterior pole of crystalline lens and horizontal line joining two scleral spurs. AAC, acute angle closure; Corn, cornea; S, sclera; I, iris; PC, posterior chamber.

Quantitative analysis by UBM and gonioscopy demonstrated a narrower anterior segment (ACD, AOD 500, and gonioscopic grading) in both eyes of the AAC group (Table 3). By analyzing the 30 patients in the AAC group, we found that the central ACD of the affected eyes was significantly shallower than that in the fellow eyes (1.82 ± 0.60 *μ*m versus 2.58 ± 0.69 *μ*m, *P* < 0.001). As for the peripheral anterior chamber, AOD 500 demonstrated a substantial decrease in the affected eyes, compared to the fellow eyes (0.06 ± 0.03 mm versus 0.25 ± 0.11 mm, *P* < 0.001). Moreover, the LV in the affected eyes was remarkably higher than in the fellow eyes (1.60 ± 0.11 mm versus 0.68 ± 0.25 mm, *P* < 0.001). However, there was no significant difference in AL, I-curve, or IT 750 when comparing affected eyes and fellow eyes in the AAC group (*P* > 0.1). As shown in Figure 1, the eye with the lower modified Shaffer grade showed a shallower ACD, smaller AOD 500, and higher LV than the fellow eye. However, there was no statistical difference among these parameters in comparisons of the affected eyes and the fellow eyes in the non-AAC group.

**Figure 2:**
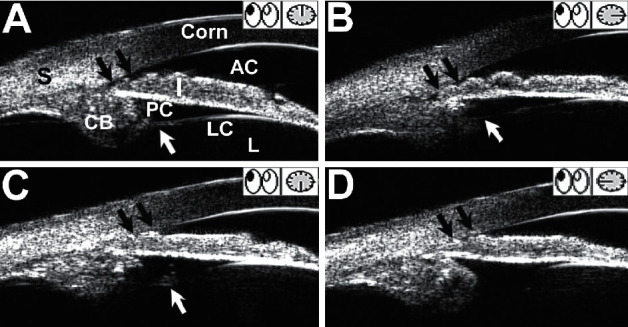
Ultrasound biomicroscopic examination of the affected eye in a patient with subluxated lens-induced AAC. (a) and (b) illustrated the structures of zonular bundles (white arrow) and slit-like opened anterior chamber angles (blank arrows) at 12 : 00 and 3 : 00, respectively. (c) An irregular hyperreflectivity (white arrow) and a completely occluded angle (blank arrows) were noted at 6 : 00, but the appearance of zonular fibers was defective. (d) The zonules were also absent at 9 : 00 and the angle was closed appositionally (blank arrows). AAC, acute angle closure; Corn, cornea; S, sclera; AC, anterior chamber; I, iris; PC, posterior chamber; L, lens; LC, lens capsule; CB, ciliary body.

By comparing the AAC group and the non-AAC group, we found that both affected and fellow eyes in the AAC group showed shallower ACD, smaller AOD 500, narrower anterior chamber angle, and shorter AL than those in the non-AAC group (*P* < 0.01, Table 3). The affected eyes of the AAC group and the non-AAC group showed significant differences in ACD, anterior chamber angle grading, AOD 500, LV, and AL (*P* < 0.01, Table 3), demonstrating that shallower ACD, smaller AOD 500, higher LV, and shorter AL are significantly associated with the secondary AAC attack. A typical case presented zonular defects at 6 : 00 and 9 : 00 position, associated with a completely occludable angle (blank arrows in Figure 2(c)–2(d)). These features were not observed in their fellow eyes and in the control eyes.

## 4. Discussion

The systemic anomalies which could cause lens subluxation such as Marfan syndrome, Weill-Marchesani syndrome, and homocystinuria were excluded from the present study. Moderate or severe lens subluxation could lead to a deepening of the anterior chamber with posterior dislocation of the crystalline lens, which was also excluded from this study. Only patients with mild lens subluxation were included in our study, and the zonular compromise between the two groups was comparable (Figure 2). Mild lens subluxation could result in undetectable clinical manifestation or slight refractive error which can be corrected with glasses. Anterior dislocation of the lens could cause pupillary block, secondary angle closure, and IOP rise, but not all lens subluxation can result in secondary glaucoma. For the patients with Marfan syndrome, even subluxated lens movement anteriorly, the pupillary block, and angle closure rarely happened due to lens subluxation [11]. The shallow anterior chamber, thick lens, anterior lens position, and short AL are important anatomical features for the primary angle closure glaucoma [12]. In this study, we compared the patients with mild lens subluxation having acute angle closure history or not and investigated ocular anatomic differences in anterior segment structure, ACD, and AL between them.

Lens subluxation may be acquired due to blunt external trauma, iatrogenic zonular damage, or uncertain etiology [13]. Our study showed that 73.3% of patients in the AAC group and 70% patients in the non-AAC group had a history of blunt ocular trauma, and eyes with AAC attack had a longer duration of prior ocular trauma histories than those in the non-AAC group (8.73 years versus 1.04 years). Ocular blunt trauma has been reported to induce zonular damage in 42.9% of patients [14, 15]. The trauma, such as fist injury, door striking, and ball hitting, was always ignored by the patients, although the history of ocular trauma mainly depended on the memory of the patient in the AAC group. Therefore, a more careful, trauma-focused history taking was suggested to help diagnose lens subluxation-induced AAC [16]. Laser peripheral iridotomy (LPI) or surgical peripheral iridectomy (SPI) is effective to relieve the acute angle closure in primary AAC when pupillary block occurs, but LPI or SPI is rarely effective in IOP reduction for patients with AAC secondary to lens subluxation [7, 17]. Lens subluxation-induced angle crowding can lead to acute or chronic angle closure despite a patent peripheral iridectomy [18]. In this study, fifteen eyes (50%) in the AAC group had an LPI or SPI treatment history when they suffered previous acute attacks, but high pressure or an AAC attack recurred. For the patients with acute angle closure caused by lens subluxation, IOP can be controlled effectively only by removal of dislocated lens. For the patients in the non-AAC group, lens extraction can improve the visual acuity, and the IOP has no difference before and after cataract surgery.

Anterior chamber asymmetry is an important clinical manifestation of lens subluxation but could be neglected due to acute attack [16]. The difference of anterior chamber depth between the affected eye and the fellow eye was detected in both groups by UBM. Our results showed that the average central ACD of affected eyes in the AAC group was 1.82 mm, which was significantly shallower than the fellow eyes (2.58 mm) or the eyes in the non-AAC group. Previously, studies of eyes with AAC secondary to lens subluxation found that the ACD of the affected eyes was significantly shallower than that of their fellow eyes (1.34 mm versus 2.27 mm and 1.29 mm versus 2.12 mm, respectively), consistent with the results in our present study [16, 19]. The central ACD of affected eyes in the non-AAC group was slightly shallow, but there was no statistical difference compared with the fellow eyes. Furthermore, AOD 500 and LV also showed remarkable differences between the affected eyes and the fellow eyes in the AAC group. Anteriorly positioned crystalline lens was showed by an LV increase in the eyes of AAC group. A higher LV may have a predominant role in AAC attack [20]. These results indicated that lens subluxation-induced AAC gave rise to greater central and peripheral ACD differences between affected and fellow eyes. The asymmetry of anterior chamber was an important feature of lens subluxation-induced AAC and could be observed by slit-lamp microscopy.

In addition, the eyes of the AAC group showed significantly shorter axial length than those in the non-AAC group. Short axial length (AL) is considered as one of the predisposing factors for development of angle closure glaucoma [21]. A subluxated or forward-tilted lens against the iris could cause a shallow anterior chamber, but a crowded anterior chamber and shorter AL might be an anatomic basis for the eye with AAC caused by lens subluxation. Compared with the eyes in AAC group, the eyes in the non-AAC group had deeper anterior chamber, longer AL, and wider chamber angle; therefore, they were less likely to have acute angle closure.

## 5. Conclusions

Mild lens subluxation may have completely different clinical manifestations in the eyes with different anatomic structure. An asymmetrical anterior chamber between bilateral eyes is a representative and important feature in lens subluxation-induced AAC. Quantitative evaluation of the ocular structure to identify the symmetry of anterior chamber, zonular defects, and lens vault increase by UBM is valuable for the diagnosis of secondary AAC due to lens subluxation. The crowded anterior chamber structure and shorter AL might be an anatomic basis for the eyes with AAC caused by lens subluxation compared with the eye without AAC attack.

## Figures and Tables

**Table 1:** Comparison of demographic and clinical data of the participants.

Variables	AAC	Non-AAC	*P*-value
Cases (*n*, eyes)	30	30	—
Age (years)	60.80 ± 5.94	57.73 ± 10.18	0.073
Sex (number M/F)	16/14	18/12	0.605
History of blunt ocular trauma (*n*)	22 (73.3%)	21 (70%)	0.795
Duration of prior ocular trauma (years)	8.73 ± 7.46	1.04 ± 1.24	0.001
Misdiagnosed as primary AAC (*n*)	20 (66.7%)	0	—
Intervention with LPI or SPI (*n*)	15 (50%)	0	—
Use of antiglaucoma medication (*n*, pre-op)	30 (100%)	0	—
Use of antiglaucoma medication (*n*, post-op)	0	0	—

Data shown are presented as mean ± standard deviation (SD), analyzed with the Mann–Whitney *U* test and Student's *t*-test. *P*-value: AAC group versus non-AAC group. AAC, acute angle closure; *n*, number; LPI, laser peripheral iridotomy; SPI, surgical peripheral iridectomy; M/F, male/female; —: not applicable; pre-op, preoperative; post-op, postoperative.

**Table 2:** Comparison of visual acuity and intraocular pressure in the affected eyes with lens subluxation.

Parameters	AAC group (*n* = 30)			Non-AAC group (*n* = 30)		
	Preoperative	Postoperative	*P -*value	Preoperative	Postoperative	*P -*value
Visual acuity						
UDVA (decimal value)	0.28 ± 0.24	0.39 ± 0.24	0.012	0.17 ± 0.24	0.39 ± 0.27	0.0001
CDVA (decimal value)	0.41 ± 0.32	0.58 ± 0.31	0.001	0.36 ± 0.29	0.55 ± 0.31	0.001
SE (D)	0.63 ± 0.83	−0.19 ± 1.15	0.001	−3.18 ± 3.11	−0.74 ± 1.38	0.002
IOP (mmHg)	41.93 ± 13.64	14.49 ± 6.03	0.0001	14.88 ± 5.32	14.31 ± 4.63	0.310

Data shown are presented as mean ± SD, analyzed with Student's *t*-test. *P*: preoperative versus postoperative. UDVA, uncorrected distance visual acuity; CDVA, corrected distance visual acuity; SE, spherical equivalent; IOP, intraocular pressure; D, diopters; AAC, acute angle closure; *n*, number.

**Table 3:** Comparison of ocular parameters of the participants included in this study.

Parameters	AAC group (*n* = 30)			Non-AAC group (*n* = 30)			2^*P*^-value	3^*P*^-value
	Affected eyes	Fellow eyes	1^*P*^-value	Affected eyes	Fellow eyes	1^*P*^-value		
Ultrasound biomicroscopy								
ACD (mm)	1.82 ± 0.60	2.58 ± 0.69	0.0001	3.63 ± 0.94	3.91 ± 0.91	0.078	0.0001	0.0001
1AOD500 (mm)	0.06 ± 0.03	0.25 ± 0.11	0.0001	0.50 ± 0.08	0.49 ± 0.11	0.525	0.0001	0.0001
LV (mm)	1.60 ± 0.11	0.68 ± 0.25	0.0001	0.38 ± 0.11	0.40 ± 0.10	0.623	0.0001	0.0001
I-curve (mm)	0.19 ± 0.06	0.19 ± 0.08	0.845	0.17 ± 0.06	0.19 ± 0.04	0.151	0.121	0.814
IT750 (mm)	0.38 ± 0.04	0.37 ± 0.07	0.423	0.37 ± 0.05	0.36 ± 0.05	0.390	0.513	0.391
Zonular compromise (laxity or loss) (*n*)	30	0	0.0001	30	0	0.0001	1.000	1.000
1 quadrant (*n*)	24	0	0.0001	22	0	0.0001	0.549	1.000
2 quadrants (*n*)	6	0		8	0			
≥3 quadrants (*n*)	0	0		0	0			
Gonioscopy								
Modified Shaffer grade, 0–4	1.57 ± 0.62	3.13 ± 0.58	0.0001	3.80 ± 0.41	3.59 ± 0.51	0.120	0.0001	0.0001
IOL master								
AL (mm)	23.66 ± 0.88	23.93 ± 1.30	0.334	25.88 ± 2.61	25.93 ± 2.75	0.937	0.0001	0.001
Other clinical data								
Lens nucleus opacity, LOCS III	2.16 ± 0.36	2.13 ± 0.29	0.693	2.25 ± 0.47	2.17 ± 0.33	0.429	0.441	0.680
Lens thickness	4.31 ± 0.27	4.31 ± 0.25	0.988	4.32 ± 0.27	4.31 ± 0.24	0.914	0.864	0.928
C/D ratio	0.31 ± 0.08	0.29 ± 0.08	0.348	0.28 ± 0.05	0.29 ± 0.04	0.697	0.340	0.069
RNFL thickness (*μ*m)	97.75 ± 9.33	99.59 ± 8.85	0.260	97.37 ± 7.48	98.18 ± 9.91	0.840	0.571	0.242

Data shown are presented as mean ± SD, analyzed with the Mann–Whitney *U* test and Student's *t*-test. 1^*P*^: affected eyes versus fellow eyes. 2^*P*^: AAC group versus the non-AAC group in affected eyes; 3^*P*^: AAC group versus the non-AAC group in fellow eyes. Respectively, anterior segment parameters and zonular evaluation were measured by ultrasound biomicroscopy. Axial length was measured by IOL Master. Grading of anterior chamber angle was evaluated by gonioscopy. Lens nucleus opacity and C/D ratio were observed in slit lamp. RNFL thickness was measured by optical coherence tomography. ACD, anterior chamber depth; AOD 500, angle opening distance at 500 *μ*m from scleral spur; LV, lens vault; I-curve, iris curvature; IT 750, iris thickness at 750 *μ*m from scleral spur; AL, axial length; LOCS III, Lens Opacities Classification System III; C/D ratio, cup/disk ratio; RNFL, retinal nerve fiber layer; AAC, acute angle closure; *n*, number.

## Data Availability

All data generated or analyzed during this study are available from the corresponding author upon reasonable request.

## Supplementary Materials

Supplementary Materials Supplementary Table: comparison of ocular parameters of the affected eyes of the participants with or without LPI/SPI in the AAC group.

## References

[1] Moghimi S., Zandvakil N., Vahedian Z. (2014). Acute angle closure: Qualitative and quantitative evaluation of the anterior segment using anterior segment optical coherence tomography. *Clinical & Experimental Ophthalmology*.

[2] Suwan Y., Jiamsawad S., Supakontanasan W., Teekhasaenee C. (2017). Hidden mechanisms beyond the pupillary block in acute angle closure: Ultrasound biomicroscopic study. *Clinical & Experimental Ophthalmology*.

[3] Guo S., Wagner R., Forbes B., Tannen B., Caputo A. (2010). Capsular tension ring in the management of occult lens zonular dehiscence in infantile glaucoma. *Journal of Pediatric Ophthalmology & Strabismus*.

[4] Kim J. M., Park K. H., Han S. Y. (2012). Changes in intraocular pressure after pharmacologic pupil dilation. *BMC Ophthalmology*.

[5] Hoffman R. S., Snyder M. E., Devgan U., Allen Q. B., Yeoh R., Braga-Mele R. (2013). Management of the subluxated crystalline lens. *Journal of Cataract & Refractive Surgery*.

[6] Ang L. P. K., Aung T., Chew P. T. K. (2000). Acute primary angle closure in an asian population. *Ophthalmology*.

[7] Aung T., Ang L. P., Chan S.-P., Chew P. T. K. (2001). Acute primary angle-closure: Long-term intraocular pressure outcome in Asian eyes. *American Journal of Ophthalmology*.

[8] Wang B., Sakata L. M., Friedman D. S. (2010). Quantitative iris parameters and association with narrow angles. *Ophthalmology*.

[9] Xu B. Y., Pardeshi A. A., Burkemper B. (2018). Quantitative evaluation of gonioscopic and EyeCam assessments of angle dimensions using anterior segment optical coherence tomography. *Translational Vision Science & Technology*.

[10] Nongpiur M. E., Aboobakar I. F., Baskaran M. (2017). Association of baseline anterior segment parameters with the development of incident gonioscopic angle closure. *JAMA Ophthalmology*.

[11] Esfandiari H., Ansari S., Mohammad-Rabei H., Mets M. B. (2019). Management strategies of ocular abnormalities in patients with marfan syndrome: Current perspective. *Journal of Ophthalmic and Vision Research*.

[12] Sun X., Dai Y., Chen Y. (2017). Primary angle closure glaucoma: What we know and what we don’t know. *Progress in Retinal and Eye Research*.

[13] Khokhar S., Agrawal S., Gupta S., Gogia V., Agarwal T. (2014). Epidemiology of traumatic lenticular subluxation in India. *International Ophthalmology*.

[14] McWhae J. A., Crichton A. C. S., Rinke M. (2003). Ultrasound biomicroscopy for the assessment of zonules after ocular trauma. *Ophthalmology*.

[15] Salehi-Had H., Turalba A. (2010). Management of TraumaticCrystalline lens subluxation and dislocation. *International Ophthalmology Clinics*.

[16] Luo L., Li M., Zhong Y., Cheng B., Liu X. (2013). Evaluation of secondary glaucoma associated with subluxated lens misdiagnosed as acute primary angle-closure glaucoma. *Journal of Glaucoma*.

[17] Saw S.-M., Gazzard G., Friedman D. S. (2003). Interventions for angle-closure glaucoma. *Ophthalmology*.

[18] Epstein D. L. (1982). Diagnosis and management of lens-induced glaucoma. *Ophthalmology*.

[19] Kwon J., Sung K. R. (2017). Factors associated with zonular instability during cataract surgery in eyes with acute angle closure attack. *American Journal of Ophthalmology*.

[20] Zhang X., Liu Y., Wang W. (2017). Why does acute primary angle closure happen? Potential risk factors for acute primary angle closure. *Survey of Ophthalmology*.

[21] Wright C., Tawfik M. A., Waisbourd M., Katz L. J. (2016). Primary angle-closure glaucoma: An update. *Acta Ophthalmologica*.

