# Supplementary material for: Comparisons of Ocular Anatomic Differences of Lens-Subluxated Eye with or without Acute Angle Closure: A Retrospective Study
Source: J Ophthalmol. 2020 Jul 30;2020:6974202. doi: 10.1155/2020/6974202 (PMC7415100; doi:10.1155/2020/6974202)
Supplement: Supplementary Materials — Supplementary Table: comparison of ocular parameters of the affected eyes of the participants with or without LPI/SPI in the AAC group. [file 6974202.f1.docx]

| **Supplementary Table. Comparison of ocular parameters of the affected eyes of the participants with or without LPI/SPI in AAC group.** | | | |
| --- | --- | --- | --- |
| Parameters | The affected eyes of AAC group | | |
|  | with LPI/SPI | without LPI/SPI | *P* value |
| Ultrasound Biomicroscopy |  |  |  |
| ACD (mm) | 1.96 ± 0.56 | 1.68 ± 0.62 | 0.203 |
| AOD500 (mm) | 0.07 ± 0.04 | 0.06 ± 0.02 | 0.688 |
| LV (mm) | 1.57 ± 0.11 | 1.62 ± 0.10 | 0.201 |
| I-curve (mm) | 0.18 ± 0.05 | 0.19 ± 0.05 | 0.748 |
| IT750 (mm) | 0.38 ± 0.04 | 0.37 ± 0.03 | 0.458 |
| Zonular compromise  (laxity or loss) (n) | 15 | 15 | 1 |
| 1 quadrant (n) | 11 | 13 | 0.539 |
| 2 quadrants (n) | 4 | 2 |  |
| ≥ 3 quadrants (n) | 0 | 0 |  |
| Gonioscopy |  |  |  |
| Modified Shaffer grade, 0-4 | 1.40 ± 0.69 | 1.73 ± 0.51 | 0.143 |
| IOL Master |  |  |  |
| AL (mm) | 23.71 ± 0.74 | 23.60 ± 1.02 | 0.749 |
| Other clinical data |  |  |  |
| Lens nucleus opacity, LOCS III | 2.27 ± 0.41 | 2.07 ± 0.26 | 0.128 |
| Len thickness | 4.31 ± 0.28 | 4.31 ± 0.27 | 0.948 |
| C/D ratio | 0.36 ± 0.07 | 0.29 ± 0.09 | 0.316 |
| RNFL thickness (μm) | 96.71 ± 4.93 | 98.90 ± 6.50 | 0.345 |
| Data shown is presented as mean ± SD, analyzed with the Mann-Whitney U test and Student’s *t*-test. *P*: affected eyes vs. fellow eyes. ^2^*P*: AAC group vs. the non-AAC group in affected eyes; ^3^*P*: AAC group vs. the non-AAC group in fellow eyes. Respectively, anterior segment parameters and zonular evaluation were measured by ultrasound biomicroscopy. Axial length was measured by IOL Master. Grading of anterior chamber angle was evaluated by gonioscopy. Lens nucleus opacity and C/D ratio were observed in slit lamp. RNFL thickness was measured by optical coherence tomography. ACD, anterior chamber depth; AOD 500, angle opening distance at 500 μm from scleral spur; LV, lens vault; I-curve, iris curvature; IT 750, iris thickness at 750 μm from scleral spur; AL, axial length; LOCS III, Lens Opacities Classification System III; C/D ratio, cup/disk ratio; RNFL, retinal nerve fiber layer; AAC, acute angle closure; n, number. | | | |
